# Rapid determination of domoic acid in seafood by fluorescence polarization immunoassay using a portable analyzer

**DOI:** 10.1007/s44211-023-00413-6

**Published:** 2023-08-31

**Authors:** Yu Ogura, Mao Fukuyama, Motohiro Kasuya, Koji Shigemura, Sergei A. Eremin, Manabu Tokeshi, Akihide Hibara

**Affiliations:** 1 Institute of Multidisciplinary Research for Advanced Materials, Tohoku University, Sendai, Japan; 2 Faculty of Production Systems Engineering and Sciences, Komatsu University, Komatsu, Japan; 3 Tianma Japan, Ltd., Kawasaki, Japan; 4 Bach Institute of Biochemistry, Research Centre of Biotechnology, Russian Acad. Sci, Moscow, Russia; 5 Division of Applied Chemistry, Faculty of Engineering, Hokkaido University, Sapporo, Japan; 6 Present Address: Departmentof Chemistry, School of Science, Tokyo Institute of Technology, Tokyo, Japan

**Keywords:** Phycotoxin, Immunoassay, Shellfish poisoning, Onsite analysis

## Abstract

Monitoring phycotoxin accumulation in marine products such as edible shellfish is a regulatory requirement in many countries. Therefore, a simple and rapid onsite quantification method is sought. Herein, we present a fluorescence polarization immunoassay (FPIA), a well-known one-step immunoassay, using a portable fluorescence polarization analyzer for domoic acid (DA), widely referred to as the primary toxin of amnesic shellfish poisoning (ASP). To establish FPIA for DA, the matrix effect of methanol, which is widely used to extract DA from shellfish, on FPIA was investigated. To validate this method, we performed a spike recovery test using oysters containing DA at a concentration equivalent to the regulatory limits of North America and the European Union (20 mg/kg). The recovery rate was found to be 79.4–114.7%, which is equivalent to that of the commercially available enzyme-linked immunosorbent assay (ELISA). We expect that this FPIA system will enable the quantitative onsite analysis of DA and significantly contribute to the safety of marine products.

**Graphical Abstract:**

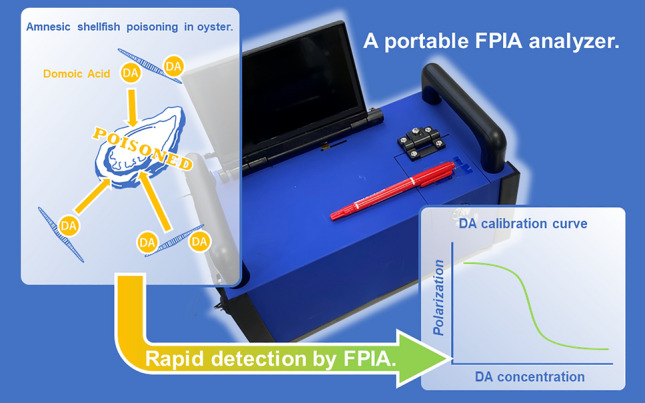

**Supplementary Information:**

The online version contains supplementary material available at 10.1007/s44211-023-00413-6.

## Introduction

**Fig. 1:**
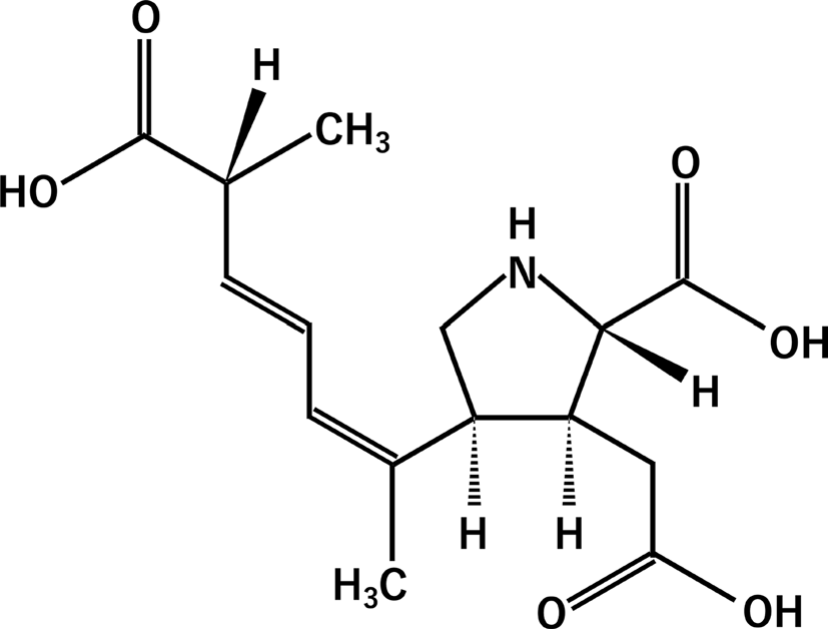
Molecular structure of domoic acid (DA)

Many of the marine toxins are phycotoxins produced by phytoplankton, and they accumulate in shellfish tissues through biomagnification in the food chain [1]. Shellfish poisoning caused by marine toxins includes paralytic shellfish poisoning, diarrheal shellfish poisoning, and amnesic shellfish poisoning (ASP). These are serious threats to public health and fisheries in temperate to cold waters [1–3]. Domoic acid (DA; Fig. 1), widely referred to as the primary toxin of ASP, is produced by marine algae. DA is produced by the diatom the genus *Pseudo-nitzschia*, a type of phytoplankton that accumulates in bivalve shellfish [1–3]. The regulatory limit for DA in seafood in North America and the European Union is 20 mg/kg [1, 4].

For the detection of marine toxins contained in seafoods, historically, an animal-based assay was used. However, it was replaced by chemical assays because the animal-based method not only has ethical problems but also is expensive and has low reproducibility [1, 4] For DA detection, high-performance liquid chromatography (HPLC) was widely adopted as an official method [1, 5, 6]. However, this method has drawbacks such as the requirement of skilled personnel and expensive and large-scale systems. A mass spectrometry method was developed from the perspective of improving analytical throughput [7]; however, this technique also requires expensive systems and skilled personnel.

**Fig. 2:**
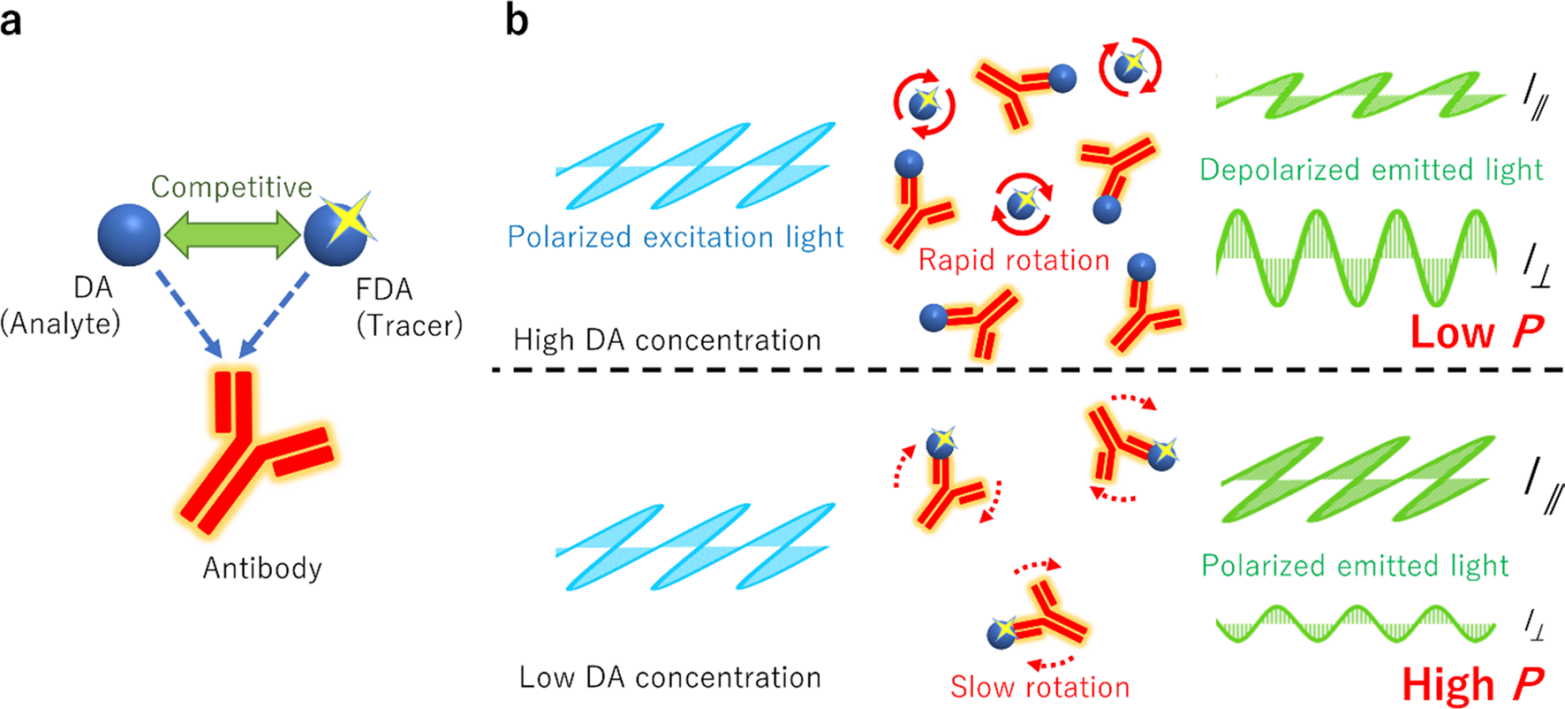
Schematic illustration of DA quantification using the fluorescence polarization immunoassay (FPIA). **a** Domoic acid (DA) and fluorescein isothiocyanate-labeled DA (FDA; tracer) bind to anti-DA antibody competitively. **b** When the DA concentration is high (top), FDA is predominantly free, resulting in low *P*. When the DA concentration is low (bottom), FDA binds to the antibody, resulting in high *P*

**Fig. 3:**
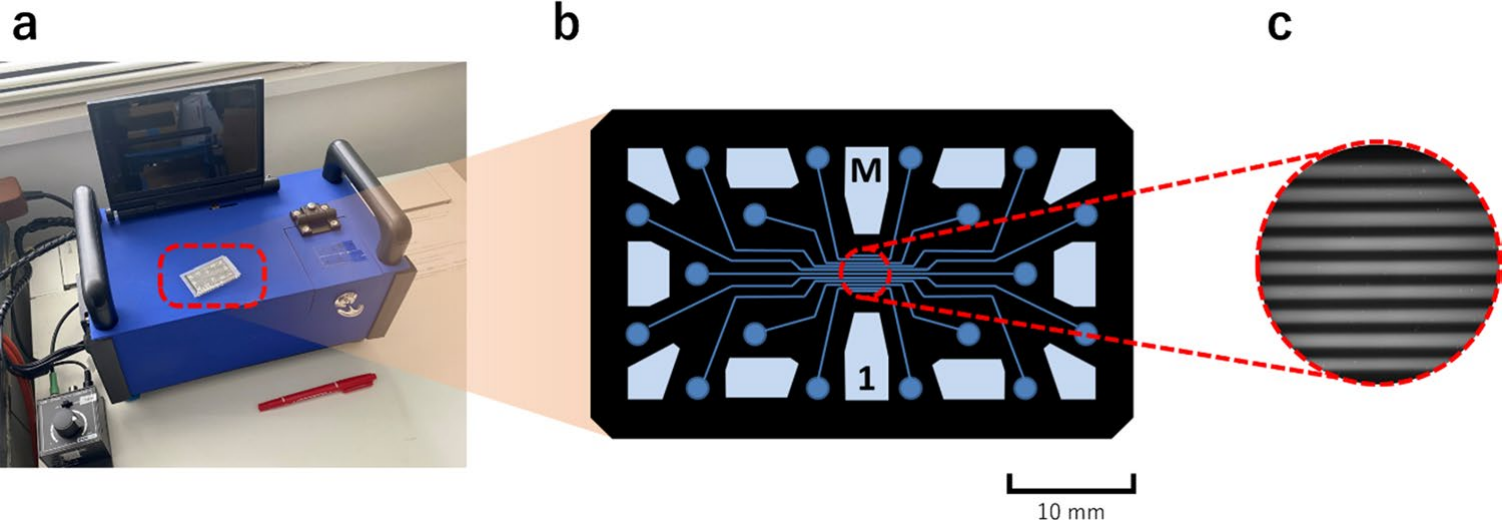
Portable FPIA analyzer. **a** Photograph of the potable analyzer and a microfluidic device (red box). **b** Channel layout of the microfluidic device. **c** Typical fluorescence image obtained using the portable analyzer

For improving cost-effectiveness in terms of the time, delivery, and human resources, a simple and rapid onsite quantification method for DA is desired. Accordingly, various immunoassays have been developed [8, 9]. For example, an enzyme-linked immunosorbent assay (ELISA) was developed for DA quantification [8, 10, 11] and ELISA kits are currently commercially available [12]. Although ELISA has high sensitivity and enables quantification, it requires multiple washing cycles, which renders the operation complicated and time-consuming. Recently, a lateral-flow immunoassay was reported for DA detection [9, 13]. Although this method has a high potential on the rapid detection of DA, quantitative portable analysis has not been demonstrated. Herein, we present a fluorescence polarization immunoassay (FPIA) that enables rapid quantification of DA with a portable analyzer. The FPIA is a well-known single-step immunoassay in which the analyte is quantified by measuring the degree of fluorescence polarization (*P*) [14, 15]. This method is based on the competitive binding of the analyte (antigen) and a fluorescence-labeled analyte (tracer) to the antibody (Fig. 2a). The ratio of the free tracer to bound tracer reflects the analyte concentration, which can be determined from the degree of *P*. When polarized light is used for excitation, the smaller free molecule emits depolarized fluorescence owing to its fast rotational diffusion, resulting in low *P*, whereas the larger bound tracer exhibits the opposite tendency (Fig. 2b). Recently, we developed a portable FPIA analyzer for the onsite analysis of analytes (Fig. 3) [16, 17]. The potential of this analyzer for onsite analysis has been demonstrated by the rapid measurement protein biomarkers [18, 19] and viruses in serum [20]. We have reported that this analyzer can be used for the detection of mycotoxins in wheat-based foods which are pretreated by using the conventional extraction method [21]. However, the matrix effects of animal tissues such as shellfish have not been investigated yet. In this study, we established a procedure for DA quantification using the FPIA and demonstrate a spike-recovery test for DA present in oysters using the portable analyzer.

## Materials and methods

### Reagents

Methanol (CH_3_OH) and phosphate-buffered saline (PBS) were purchased from FUJIFILM Wako Pure Chemical Corp. (Osaka, Japan). Domoic Acid (DA) was obtained from Toronto Research Chemicals Inc. (Toronto, Canada). Fluorescein isothiocyanate-labeled DA (FDA) was synthesized by SCRUM Inc. (Tokyo, Japan), according to a previously reported procedure [20]. The EuroProxima Domoic acid ELISA kit was obtained from R-Biopharm Nederland B.V. (Arnhem, The Netherlands).

### Measurement of the degree of fluorescence polarization (P) using the portable FPIA analyzer

We measured *P* using a portable FPIA analyzer that was previously developed in our groups (Fig. 3a) and disposable microfluidic devices with nine microchannels (Fig. 3b and c) [16, 17]. The microfluidic device is made of black poly (dimethylsiloxane) and a glass plate. Typically, 20–30 µL of sample solutions were injected into the nine channels of the microfluidic device (width 200 µm, height 900 µm) by a 100 µL-sized micropipette, and the fluorescence from the samples in the microchannels was detected simultaneously using a CCD camera (Fig. 3c) to evaluate the *P* values of nine samples independently.

### Preparation of FDA dilution series and verification of optimal concentration

Aqueous solutions of FDA in PBS were prepared at 0.143–14,300 nM concentrations. Each solution was injected into the microchannels in the device, and the *P* of each solution was measured using the portable analyzer.

### Verification of the effect of methanol on P measurement

Samples containing FDA (1.43 nM), DA (0 nM or 64 nM), 0.00–0.13% (v/v) methanol, and anti-DA antibodies were prepared in PBS. As an anti-DA antibody, we used the antibody in the DA ELISA kit. After incubation at room temperature for 1 h, the samples were injected into the microfluidic device and *P* was measured using a portable analyzer.

### Addition and extraction of DA in shellfish for spike-recovery tests

Raw edible oysters (*Crassostrea gigas*) were used as an example of shellfish. The soft body of the oyster was ground using an electric hand blender and processed into a paste. Next, 100 µL of a 400 µg/mL DA solution was added to 2.0 g of the shellfish paste, and the resulting mixture was placed in a 15 mL test tube and mixed by tapping. After 30 min of incubation at room temperature, DA was extracted from the oyster paste using the methanol extraction method [4, 22] adopted in the approved official method [6]. For this, 2 mL of Milli-Q water was added to the oyster paste and the mixture was vortexed for 1 min. Then, 4 mL of methanol was added, and the mixture was vortexed for 3 min. Finally, the mixture was centrifuged at 2000 × g for 10 min, and the supernatant was collected and filtered through a Millipore® Millex® syringe filter (0.45 µm pore size; Merck KGaA, Darmstadt, Germany). The filtration using the syringe filter takes 3 min. A portion of the supernatant of the extract was dialyzed using Millipore^®^ Amicon^®^ Ultra (nominal molecular weight cutoff: 10 kDa, Merck KGaA, Darmstadt, Germany) to eliminate macromolecular contaminants (30 min).

### Spike-recovery test using the FPIA

A calibration curve of DA was constructed using standard solutions containing 0.26 nM to 64 nM of DA, 1.43 nM FDA, anti-DA antibody, and 0.01% (v/v) methanol in PBS. As an analyte solution, a mixture of the oyster extract diluted by 5,000-fold, 1.43 nM FDA, anti-DA antibody, and 0.01% (v/v) methanol was prepared in PBS. After 10, 30, and 60 min of incubation, the solutions were injected into the microfluidic device and *P* was measured using the portable analyzer. The limit of detection (LOD) was calculated as the DA concentration corresponding to the *P*_LOD_ = *P*_ave_ + 3* s*, where *P*_ave_ and *s* are the mean and standard deviation of *P* with the lowest DA concentration (0.143 nM), respectively [19].

### Spike-recovery test using ELISA

DA in the oyster extract was also determined using the ELISA kit, according to the manufacturer’s instructions.

## Results and discussion

**Fig. 4:**
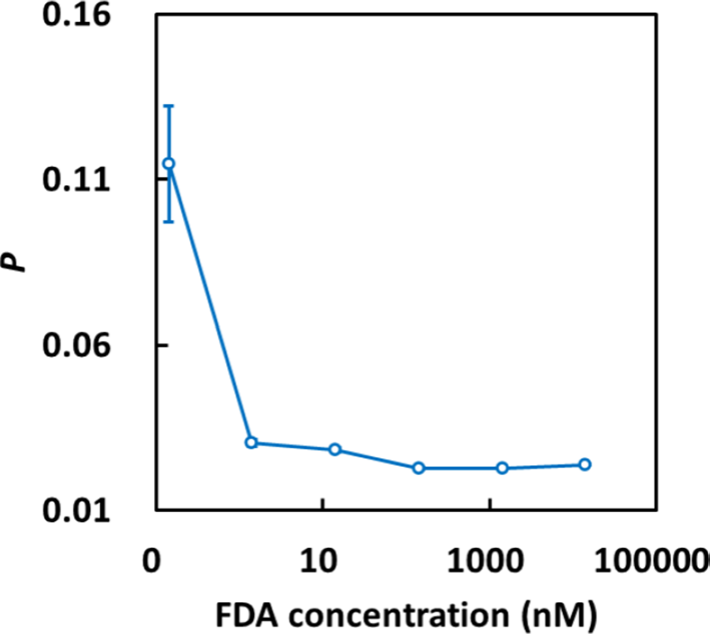
Dependence of *P* on the FDA concentration

First, the *P* values of FDA aqueous solutions with different FDA concentrations were evaluated by the FPIA using a portable analyzer. In this experiment, *P* should be constant regardless of the FDA concentration, because the solution contains only free FDA molecules. However, as shown in Fig. 4, the value of *P* was constant above 1.43 nM, but increased below 1.43 nM. In the low-concentration condition, certain interfering factors, such as the fluorescence of PDMS and the adsorption of FDA to the microchannel walls, may affect the *P* value. Since the lower tracer concentration is better from the viewpoints of the sensitivity of FPIA and the consumption of the antibody, we chose to use 1.43 nM FDA in subsequent experiments. The dilution factor of the antibody was optimized by using 1.43 nM FDA.

**Fig. 5:**
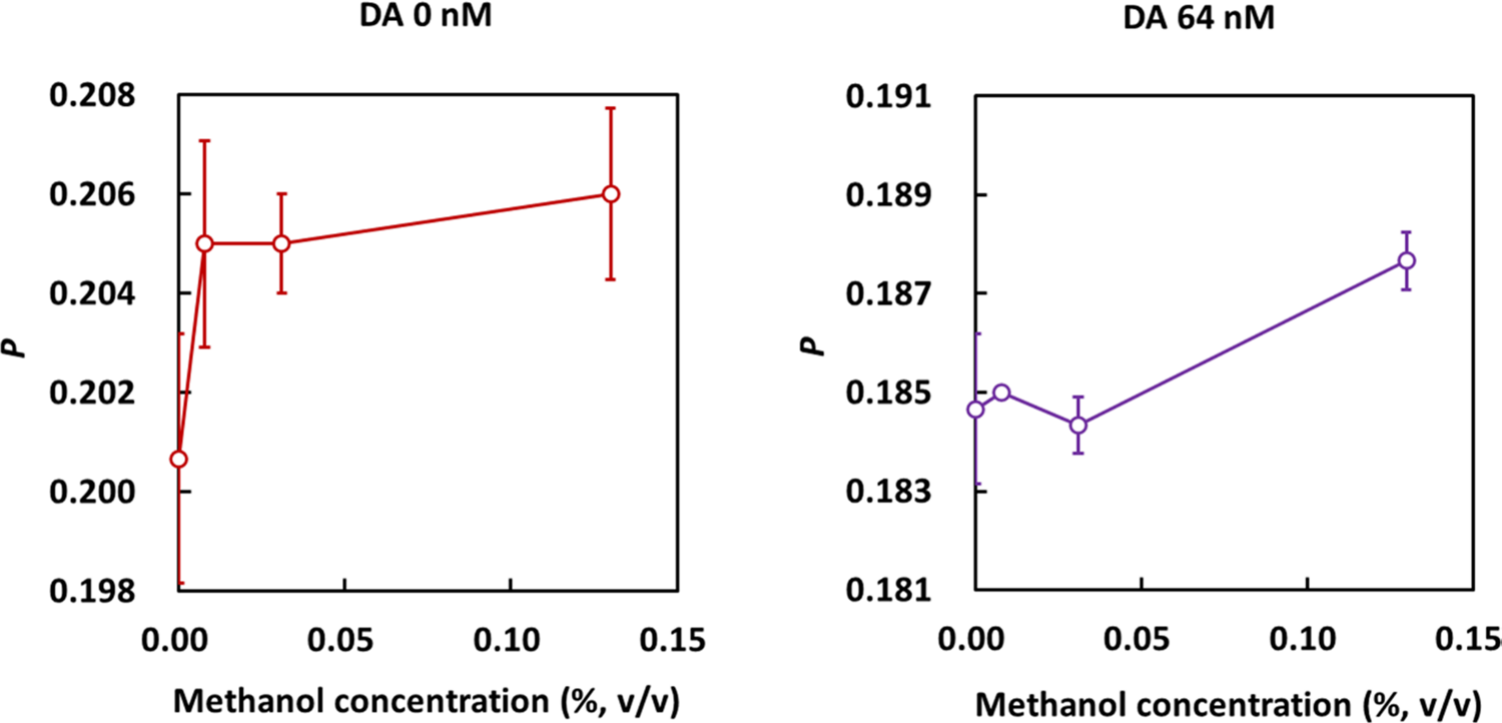
Dependence of *P* on methanol concentration in FPIA solutions. The solutions contained methanol, 1.43 nM FDA, anti-DA antibody, DA (0 nM **a** or 64 nM **b**), and PBS

Next, we confirmed the effect of methanol addition on the FPIA, because methanol is commonly used to extract DA from shellfish in the official methods of DA quantification [4, 22]. As shown in Fig. 5, although the P value of tracer-antibody complex (DA 0 nM) is higher than that of free tracer (DA 64 nM), which indicates the antibody can bind to the tracer with the existence of methanol, the addition of methanol increased the *P* value. One possible reason of P increase is that the viscosity of the methanol–water mixture in low methanol concentration range is slightly higher than that of pure water [23] even though the viscosity of pure methanol is lower than that of pure water. For DA detection, methanol was added to the standard solutions used for constructing the calibration curve to compensate for the matrix effect.

**Fig. 6:**
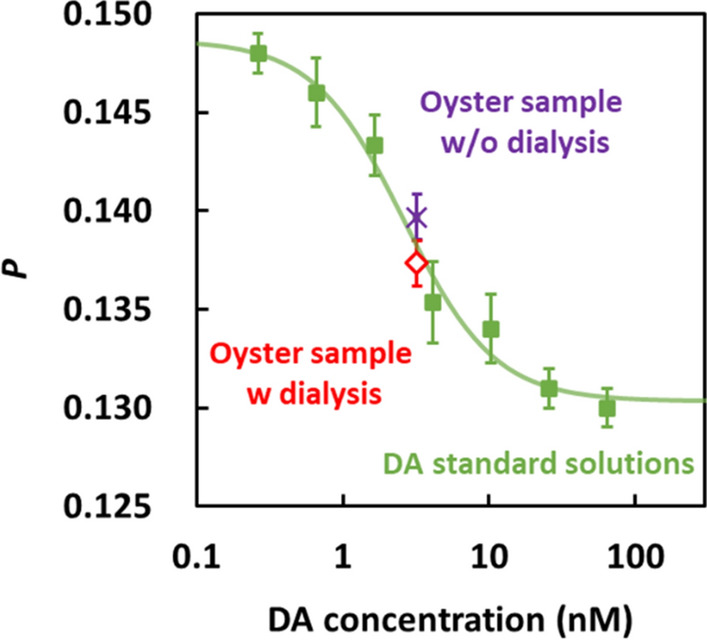
Quantification DA using the FPIA. The symbols represent the calibration curve constructed using standard DA solutions (green) and the DA concentration in oyster extracts processed with (red) and without (purple) dialysis. LOD was calculated as 0.97 nM from the calibration curve

Based on the above investigations, a spike-recovery test of DA in oysters was performed. Nine solutions, including seven DA standard solutions and two oyster extract samples, were analyzed at the same time using the microfluidic device with nine microchannels. A calibration curve was obtained for *P* using the standard solutions of DA (Fig. 6). The limit of detection (LOD) was calculated as 0.97 nM by fitting the sigmoidal function by following the previous study [19]. Oyster-based samples prepared with and without dialysis were evaluated because FPIA is sometimes affected by contaminant proteins in the sample solution. The DA concentration of the oyster extract was determined before and after dialysis using the calibration curve. The DA concentrations in the respective samples were 3.67 nM (22.8 mg/kg of oyster, recovery, 114.7%) and 2.54 nM (15.8 mg/kg of oyster, recovery, 79.4%), respectively (Table 1). These results reveal that FPIA can quantify DA, regardless of the dialysis of the oyster extract. Investigation of the reaction time indicated that a reaction time longer than 20 min was sufficient for DA quantification (Figure S1). Since the accuracy of FPIA is not significantly different with and without dialysis, dialysis can be omitted to reduce analysis time. A comparison of the results of FPIA and ELISA indicated that the recovery rates of the two assays are comparable (Table 1). In addition, the comparison with other DA quantification methods revealed that this method achieves both high portability and short measurement time (Table S1). This result indicates the potential applicability of FPIA as an onsite method for the quantification of DA in the future. We expect that this FPIA portable analyzer will be applied to other phycotoxin detection using FPIA [24, 25].

**Table 1:** Comparison of DA quantification using FPIA and ELISA

Method	Spiked DA (mg/kg)	Found (mg/kg)	Recovery (%)
FPIA (with dialysis)	20.0	22.8	110.9 ± 11.0
FPIA (without dialysis)	20.0	15.8	80.6 ± 11.3
ELISA	20.0	17.3	86.3 ± 2.3

## Conclusion

In this study, we developed a DA quantification method using a portable FPIA analyzer. The investigation of the matrix effect of the methanol–water mixture showed that P increased with the addition of methanol and matrix matching was required for FPIA. A Spike recovery test of DA in oysters indicated that the developed FPIA could quantify DA with recovery similar to that of commercial ELISA. This method is expected to be useful in the onsite quantification of DA because of the simple operation and portability of the equipment. We expect that the cost of this immunoassay method will be similar to an ELISA kit ($10 ~ 100 /test) in the future. Compared to LFIA, which is a typical portable on-site testing, this method is expected to be more quantitative and reproducible. In addition, since FPIA is a one-step homogeneous immunoassay, it requires less expertise than other multistep immunoassays such as ELISA and other instrumental analyses such as HPLC and LC-mass spectrometry. Therefore, we consider that this method will be a powerful tool for on-site analysis of DA and will enable us to reduce various costs such as labor and transportation.

### Supplementary Information

Below is the link to the electronic supplementary material.

Supplementary file1 (PDF 444 KB)

## Data Availability

The datasets generated during and/or analyzed during the current study are available from the corresponding author on reasonable request.
