# Supplementary material for: Rapid determination of domoic acid in seafood by fluorescence polarization immunoassay using a portable analyzer
Source: Anal Sci. 2023 Aug 31;39(12):2001–6. doi: 10.1007/s44211-023-00413-6 (PMC10667144; doi:10.1007/s44211-023-00413-6)
Supplement: Supplementary file 1 — Supplementary file1 (PDF 444 KB) [file 44211_2023_413_MOESM1_ESM.pdf]

Supporting Information for

Rapid determination of domoic acid in seafood by fluorescence polarization immunoassay using a portable analyzer

Yu Ogura, Mao Fukuyama*, Motohiro Kasuya, Koji Shigemura, Sergei A. Eremin, Manabu Tokeshi, Akihide Hibara*

Corresponding authors:
Mao Fukuyama and Akihide Hibara
maofukuyama@tohoku.ac.jp, Institute of Multidisciplinary Research for Advanced Materials, Tohoku University, Sendai, Japan
hibara.a.aa@m.titech.ac.jp, Institute of Multidisciplinary Research for Advanced Materials, Tohoku University, Sendai, Japan

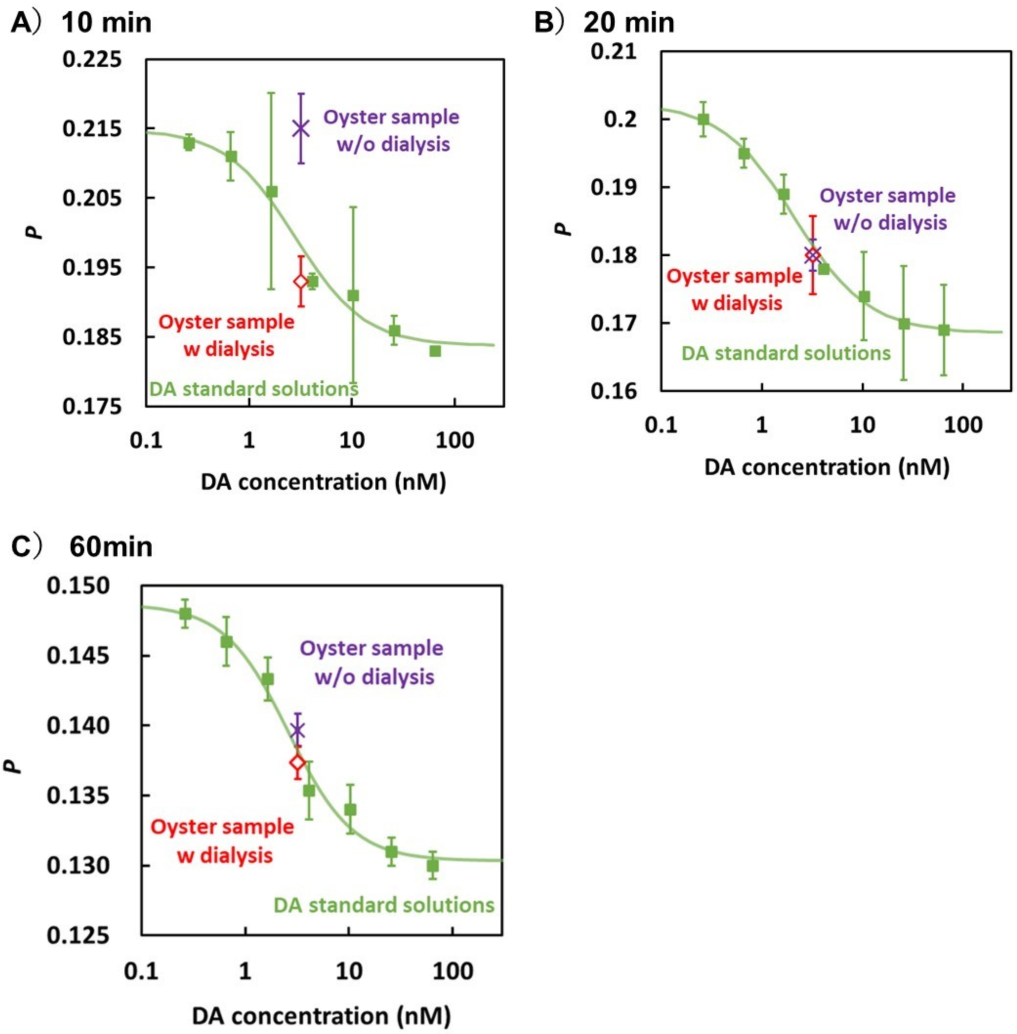

Fig. S1 Investigation of reaction time. (A) 10 min, (B) 20 min, and (C) 60 min.

Table S1 Comparison of DA quantification methods.

Method	Measurement time (min)	Sample amount (g)	Apparatus size (cm)	Apparatus weight (kg)	Portability	LOD(M)	Quantitative
**ELISA[a]**	> 60	1	40×30×20 [c]	10 [c]		1.7×10^{-10}	✓
**HPLC[1]**	5-25[b]	100	c.a.50×50×100 [e]	90 [e]		1.9×10^{-7}	✓
**LAESI-MS[2]**	-	1	Typically 100×100×100	Typically >100[i]		1.8×10^{-6}	✓
**LFIA 1 [3]**	> 10	1	22×10×24	1.6	✓	1.6×10^{-9} [d]	
**LFIA 2 [4]**	15	1	c.a. 20×50×50 [f]	> 2	potentially good but not demonstrated	4.5×10^{-9}	✓
**Microchip electrophoresis[5]**	1[g]	c.a. 1	c.a. 50×50×100 [h]	> 10 [h]		2.8×10^{-10}	✓
**FPIA(This study)**	30	2	15×35×15	5.5	✓	9.7×10^{-10}	✓

[a] EuroProxima Domoic acid ELISA kit

[b] Exclude the time for initiating HPLC setup

[c] AS-ONE MPR-A100 plate reader as an example.

[d] Quantification of DA in shellfish sample have not been discussed

[e] Agilent 1290 Infinity II LC system as an example.

[f] Include a scanner and PC

[g] Exclude the time for initiating the microchip

[h] Include a laser, computer, and power supplier

References

[1] M.A. Quilliam, P.G. Sim, A.W. Mcculloch, A.G. Mcinnes. High-performance liquid chromatography of domoic acid, a marine neurotoxin, with application to shellfish and plankton, Int. J. Environ. Anal. Chem. 36 (1989) 139–154.

[2] D.G. Beach, C.M. Walsh, P. Cantrell, W. Rourke, S. O'Brien, K. Reeves, P. McCarron, Laser ablation electrospray ionization high-resolution mass spectrometry for regulatory screening of domoic acid in shellfish, Rapid Commun. Mass Spectrom. 30 (2016) 2379–2387.

[3] Hendrickson, O. D. *et al.* Rapid detection of phycotoxin domoic acid in seawater and seafood based on the developed lateral flow immunoassay. *Analytical Methods* 14, 2446–2452 (2022).

[4] W. Jawaid, J. Meneely, K. Campbell, M. Hooper, K. Melville, S. Holmes, J. Rice, C. Elliott. Development and validation of the first high performance-lateral flow immunoassay (HP-LFIA) for the rapid screening of domoic acid from shellfish extracts, Talanta 116 (2013) 663–669.

[5] Cheng, Y., Guo, C., Zhao, B. & Yang, L. Fast analysis of domoic acid using microchip electrophoresis with laser-induced fluorescence detection. *J Sep Sci* 40, 1583–1588 (2017).
